# Irreducible anterior and posterior dislocation of the shoulder due to incarceration of the biceps tendon

**DOI:** 10.4103/0973-6042.76970

**Published:** 2010

**Authors:** Michael S. Day, David M. Epstein, Brett H. Young, Laith M. Jazrawi

**Affiliations:** Department of Orthopaedic Surgery, NYU Hospital for Joint Diseases, New York, USA

**Keywords:** Biceps tendon, computed tomography, irreducible, shoulder dislocation

## Abstract

Mechanical obstacles may infrequently impede closed reduction of anterior shoulder dislocation. Imaging techniques such as arthrography, computed tomography (CT) and magnetic resonance imaging (MRI) complement conventional radiography by allowing identification of obstacles to reduction. We present a case of irreducible anterior glenohumeral dislocation resulting from an initial anterior dislocation, converted to a posterior dislocation with an attempt at reduction, then converted back to anterior dislocation with a second reduction attempt. Soft tissue obstacles to shoulder reduction should be suspected when plain films do not identify a bony fragment as the culprit. CT and MRI are useful for identifying the cause of irreducibility and for operative planning.

## INTRODUCTION

The glenohumeral joint is the body’s most frequently dislocated joint, with over 95% of these occurring in the anterior direction.[1] Most anterior shoulder dislocations are easily reduced.[2] However, mechanical obstacles may infrequently impede closed reduction of an anterior dislocation, necessitating open reductions. Such obstacles to reduction include: interposition of the long head of the biceps tendon,[3–11] subscapularis tendon[2,12,13] or labrum;[10] impaction of the humeral head on the glenoid rim;[14–16] as well as dislocated bony fragments from the glenoid[17] or greater tuberosity.[18–20] Imaging techniques such as arthrography, computed tomography (CT) and magnetic resonance imaging (MRI) provide crucial adjuncts to conventional radiography by allowing identification of the obstacle to reduction and appropriate planning of operative intervention.[8] We present a case of irreducible anterior glenohumeral dislocation resulting from an initial anterior dislocation, which was converted to a posterior dislocation with an attempt at reduction, then converted back to an anterior dislocation with a second reduction attempt. To our knowledge, only three other case reports exist providing CT imaging with surgical correlation of an interposition of the biceps tendon between the humeral head and glenoid,[3,8,9] with none reporting this finding secondary to iatrogenic conversion of anterior dislocation to a posterior dislocation during reduction, secondary to biceps incarceration in the glenohumeral joint.

## CASE REPORT

**Figure 1:**
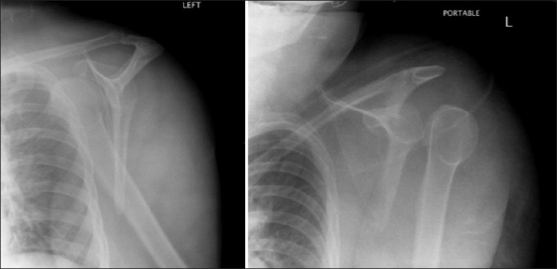
Scapular Y views of (a) anterior and (b) posterior dislocation of the left shoulder

**Figure 2:**
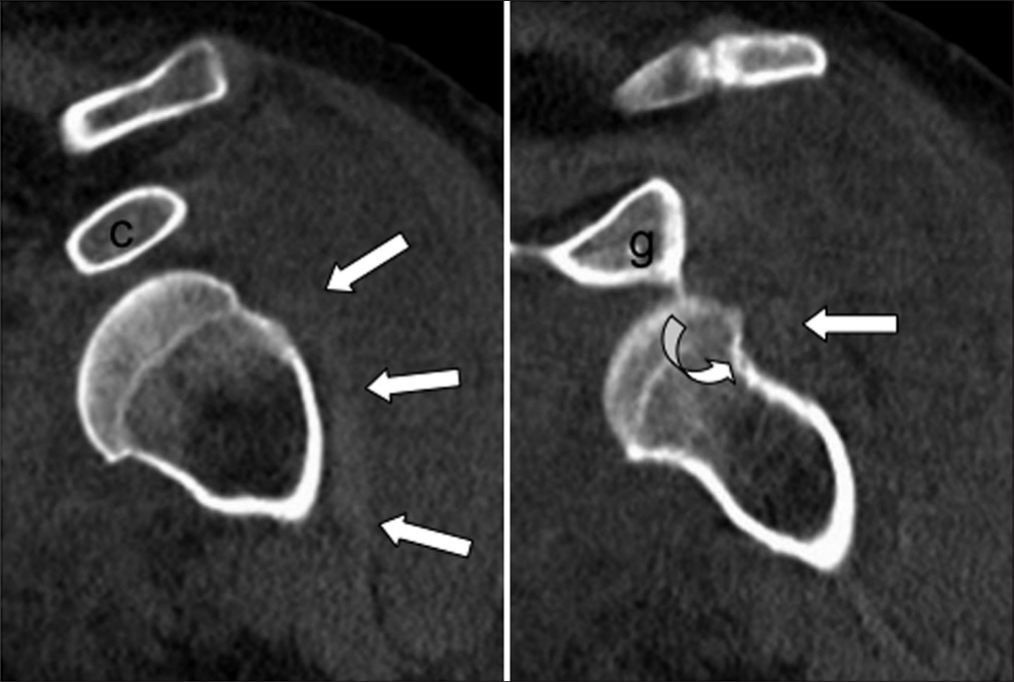
Oblique coronal 2D reconstructions demonstrate the long head of biceps tendon (white arrows) dislocated lateral and posterior to the humeral neck and head. Note a Hill–Sachs defect (curved arrow) (c: coracoid; g: glenoid)

**Figure 3:**
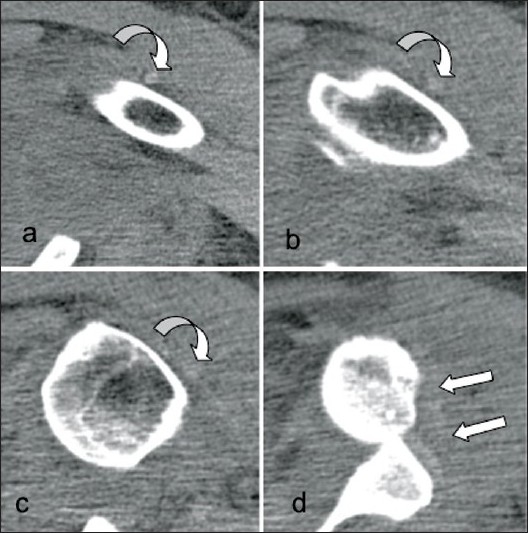
CT images obtained with axial 3 mm collimation demonstrate the long head of biceps tendon dislocated lateral to the (a) proximal humeral shaft and (b) bicipital groove as well as (c) posterior to the greater tuberosity and (d) Hill–Sachs defect. Note failed closed shoulder reduction with anterior subcoracoid dislocation of the humeral head

A 57-year-old female with mental retardation initially presented to a private hospital emergency room with complaints of left shoulder pain and decreased range of motion. The patient was a resident of a group home where she used to ambulate minimally and had no witnessed history of trauma, fall, or seizure. She had a medical history significant for mitral valve prolapse, hypercholesterolemia and bipolar disorder. Additionally, the patient had no reportable history of previous shoulder dislocation or instability. A focused history and exam at the time of presentation revealed the patient to be a poor historian given her mental retardation. On examination, she was found to be obese, with fullness about her left shoulder, and she was neurovascularly intact in the left upper extremity with limitation of range of motion in all planes. Neurologic exam and MRI were found to be negative for stroke. However, radiographic imaging of her left shoulder was found to be significant for an anterior shoulder dislocation [Figure 1a]. At this time, the patient was evaluated by an orthopedic resident and she underwent an attempted closed reduction of her left shoulder under conscious sedation. Post-reduction imaging, including plain radiographs and CT, revealed that the shoulder was now dislocated posteriorly [Figure 1b]. Again, the patient underwent a closed reduction with conscious sedation, with post-reduction radiographs revealing that the humeral head was now again dislocated anteriorly. Further attempts at reduction of this anterior glenohumeral dislocation under conscious sedation were unsuccessful. The patient was then medically cleared for surgery and repeat CT scan of the left shoulder was performed to assess for any bony defects and reasons for shoulder irreducibility. Oblique coronal and sagittal 2D reconstructions demonstrated the long head of biceps tendon dislocated lateral and posterior to the humeral neck and head. A small Hill-Sachs defect was also noted with a subcoracoid glenohumeral dislocation [Figures2 and 3].

**Figure 4:**
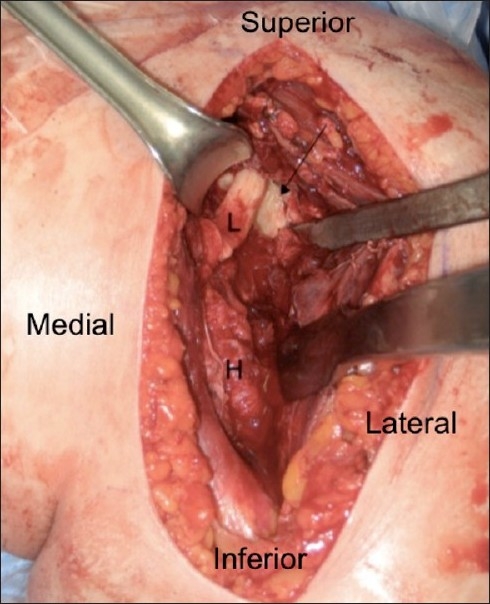
Intraoperative photo of long head of the biceps tendon (L) preventing reduction of the humerus (H) (arrow: glenoid)

At this time, the patient was brought to the operating room for attempted closed reduction under general anesthesia. After general anesthesia was administered, attempt at closed reduction was again unsuccessful and open reduction was performed. A standard delto-pectoral approach was then performed with the patient in a beach-chair position. The interval was identified, the cephalic vein retracted laterally, and dissection down to the humeral shaft was performed. The humeral head was noted to be anteriorly dislocated in a sub-coracoid position. An attempt at reduction was again unsuccessful. The biceps tendon was then identified distally in the bicipital groove and followed proximally where it was noted to be incarcerated in the glenohumeral joint. The proximal tendon was dislocated from its anatomic position in the bicipital groove proximally and was tethered laterally around the humeral head, preventing reduction of the glenohumeral joint [Figure 4]. The biceps tendon was tenotomized from its origin at the superior glenoid tubercle and the portion of interposed tendon was excised, with a soft tissue tenodesis being performed at the level of the pectoralis major tendon. Next, the subscapularis was separated from the joint capsule, which was incised and inferiorly dissected off the humeral neck. The labrum was clearly visualized and was found to be intact inferomedially. At this point, the glenohumeral joint was reduced and taken through a full range of motion of internal rotation and external rotation with the arm in adduction and at 90° of abduction. The subscapularis and capsulolabral complex was then repaired through bony tunnels in the lesser tuberosity with #2 Fiberwire (Arthrex, Naples, FL, USA). The deltopectoral interval was then closed and a standard subcuticular closure performed. Intraoperative and postoperative radiographic imaging confirmed glenohumeral reduction. This patient had an uneventful postoperative course and was discharged back to her group home with visiting nursing services on the fourth postoperative day. Immediate hand, wrist, and elbow exercises were instituted, with passive range of motion exercises instituted at 2 weeks followed by gentle active assisted range of motion exercises. Active internal rotation was limited for 4 weeks.

## DISCUSSION

A review of the literature on irreducible anterior shoulder dislocation identified only 22 cases. In nine of these cases, interposition of the long head of the biceps tendon was the obstacle to closed reduction.[3–11] In five of the nine cases, a greater tuberosity fracture allowed posterior/lateral subluxation of the biceps tendon.[5,7,8,10,11] Two of the nine cases were identified with a Hill-Sachs defect,[3,8] and three of the nine cases showed no bony lesions (other than the ectopic calcification reported by Freeland and Higgins and Rakofsky *et al*.).[4,6,9]

Our patient initially presented with an anterior dislocation which was then iatrogenically converted to a posterior dislocation with attempted reduction, typically a less common injury associated with high-energy trauma or seizure activity. Irreducible posterior dislocation has also been associated with blockage of reduction by the long head of the biceps tendon. However, our patient’s dislocation was anterior when its irreducibility became an indication for surgery. For this reason, we classified the case as one of acute irreducible anterior dislocation.

MRI would have provided better visualization of the tendon and other soft tissue structures.[2] However, in our case, CT, in addition to identifying a Hill-Sachs defect, was sensitive enough to allow visualization of the biceps tendon pathology, and was ordered to assess for bony instability as a potential cause for inability to maintain a stable reduction.

Our case represents a unique complication of attempted closed reduction of anterior shoulder dislocation. Soft tissue obstacles to successful reduction of a dislocated shoulder should be suspected when plain films do not identify a bony fragment as the culprit. CT and MRI are useful for identifying the cause of irreducibility and for operative planning.
